# Uses of Papaya Leaf and Seaweed Supplementations for Controlling Glucose Homeostasis in Diabetes

**DOI:** 10.3390/ijms24076846

**Published:** 2023-04-06

**Authors:** Benard B. Nyakundi, Jinzeng Yang

**Affiliations:** Department of Human Nutrition, Food and Animal Sciences, University of Hawaii at Manoa, Honolulu, HI 96822, USA

**Keywords:** bioactive compound, diabetes, hyperglycemia, seaweed, *Carica papaya*, type 2 diabetes, insulin resistance, herbs, secondary metabolites, natural products, glucose uptake

## Abstract

Studies from laboratory animal models and complementary medical practices have implied that nutrients from special plants or herbs contain antidiabetic, antioxidant, anti-obese, anti-hypertensive, and anti-inflammatory properties. Seaweed and tropical papaya, which are widely available in Asian and Pacific countries, have been used as home remedies for centuries. The bioactive extracts from these plants contain vitamins A, C, B and E complexes, as well as polysaccharides, phenolic compounds, essential fatty acids, flavonoids, saponins, fucoidan, and phlorotannin. In this review, the authors examine the pathogenesis of diabetes characterized by hyperglycemia due to the dysregulation of glucose homeostasis, antidiabetic/antihyperglycemic seaweed or/and papaya derived bioactive phytochemicals and their proposed mechanisms of action in the management of Type 2 Diabetes Mellitus (T2DM). The authors also propose combining papaya and seaweed to enhance their antidiabetic effects, leveraging the advantages of herb-to-herb combination. Papaya and seaweed have demonstrated antidiabetic effects through in vitro assays, cellular models, and animal studies despite the limited clinical trials. Nutraceuticals with antidiabetic effects, such as secondary metabolites isolated from seaweed and papaya, could be combined for a synergistic effect on T2DM management. However, the application of these compounds in their purified or mixed forms require further scientific studies to evaluate their efficacy against diabetes-related complications, such as hyperlipidemia, elevated free radicals, pro-inflammatory molecules, insulin insensitivity, and the degeneration of pancreatic beta cells.

## 1. Introduction

The prevalence of Type 2 Diabetes Mellitus (T2DM) has seen an exponentially high upsurge on a global scale. Over the past few decades, more than 415 million people between the ages of 20–79 years are reported to have suffered from the disease globally. It is estimated that there will be 614 million cases and more than 5 million deaths due to T2DM by 2040 [1]. T2DM is a chronic disease that reduces the immune system of the body against many infectious diseases such as coronavirus (i.e., the COVID-19 pandemic). In addition to the risk factor of advancing age for T2DM, childhood obesity has augmented T2DM incidents among adolescents and young adults, which could cause a serious public health crisis. This has resulted in strained healthcare service provision due to diabetic-associated comorbidities [2]. A variety of factors contribute to the development of type 2 diabetes, including genetics, environment, sedentary lifestyles, food intake, nutrition, and obesity. The onset of T2DM is characterized by reduced peripheral insulin sensitivity, which appears in the skeletal muscles, adipose tissues, liver, and the gradual degeneration of pancreas β cells. When insulin resistance occurs, insulin demand rises, leading to hyperinsulinemia due to the compensatory increase in both cell mass and insulin secretion. Hyperinsulinemia inflames the metabolic dysregulations, which leads to the collapsing of β-cells and the subsequent development of T2DM.

There is an indication of therapeutic effects in natural products due to various bioactive substances that regulate a broad range of metabolisms and cellular functions in the body. Marine algae-derived phytochemicals, such as sterols, proteins, sulfated polysaccharides, fatty acids, pigments, and antioxidants, have been demonstrated to have antihyperglycemic effects. In their detailed review, Zhao and colleagues discussed the bioactive compounds found in seaweed with antidiabetic effects and the ongoing preclinical trials [3]. On the other hand, papaya, as one of the most common tropical fruits with antihyperglycemic characteristics, possesses many of the proposed therapeutic effects on T2DM [4]. Combining different sources of herbs or natural products for therapeutic purposes is a common practice in traditional medical practices. This is in contrast to the classical drug discovery approach, which has focused on “One disease–one target–one drug” for a long time. However, the shifts towards polypharmacy, in which multiple drugs are given for conditions such as cancer and acquired immunodeficiency syndrome, can also be applied to natural products [5]. The combination of papaya and seaweed may enhance the efficacy of conventional diabetic treatment by strengthening and potentiating the phytochemicals derived from terrestrial and marine sources.

## 2. Pathogenesis of Type II Diabetes Mellitus (T2DM)

Among the most prevalent risk factors associated with type 2 diabetes and heart disease in the 21st century is metabolic syndrome. The risk is likely to be increased by five times for type 2 diabetes and by an approximate doubling of cardiovascular disease associated with metabolic syndrome [3,4]. Metabolic syndrome is said to be an amalgamation of metabolic abnormalities, including abdominal obesity, increased triglycerides, hypercholesterolemia, hypertension, reduced high-density lipoprotein (HDL) levels, and hyperglycemia [5,6]. Moreover, increased pro-inflammatory molecules, nonalcoholic fatty liver, hyper cholesterol-related complications, and abnormalities in the coagulation of the venous blood have been reported among individuals with metabolic syndrome [7,8,9,10,11,12]. It is now evident that the aggregation of metabolic abnormalities contributes to insulin resistance, which leads to uncontrolled blood sugar levels. Whether insulin resistance and hyperinsulinemia are precursors or spinoffs of metabolic syndrome is unclear. 

In normal physiology, the level of blood glucose is firmly regulated to reach 70–100 mg/dL in 1–3 h after a meal through specific hormones and their regulations on the carbohydrate and lipid metabolisms. The majority of postabsorptive glucose is disposed of by splanchnic tissues, such as the liver, which accounts for about 45% of glucose, gastrointestinal tissue, the main peripheral skeletal muscle tissues, and adipose tissue [13]. The release of insulin promotes glucose uptake into cells, resulting in cellular oxidation and glycogen synthesis, which prompts the blood glucose levels to return to pre-prandial levels within a short period. As the single largest organ in the body, the skeletal muscle is the major site of insulin-stimulated glucose uptake in the postprandial states in humans [14]. Based on the euglycemic hyperinsulinemia condition, the skeletal muscles dispose of 80% of the glucose uptake. In the state of physiological hyperinsulinemia (80–100 mU/mL), the leg muscle glucose uptake increases linearly with time, reaching a plateau value of 10 mg/kg leg weight per minute after 60 min. Insulin insensitivity in the organs and subsequent T2DM are linked to the failure of the insulin signaling pathways to uptake glucose to various organs or tissues. The chronic exposure of cells to high glucose levels in untreated T2DM patients may lead to the occurrence of toxicity, which may lead to sensitive neurons and heart muscle damage. Covalent attachments of glucose and its toxic derivative to large molecules, such as RNA, lipids, and proteins, can form advanced glycation products that block the insulin signaling pathways.

It is evident that insulin resistance is complicated by lipid metabolism. Elevated free fatty acids cause an accumulation of intramyocellular lipids, such as diacylglycerol (DAG) and ceramide [15]. In addition, aggregated DAG impairs the Insulin receptor substrate 1 (IRS-1) signal relay by activating serine kinases, which in turn phosphorylate serine residues. On the other hand, the action of ceramide diminishes glucose uptake by causing protein kinase B (AKT) signaling insufficiency [16]. Moreover, increased mitochondrial fatty acid oxidation creates reactive oxygen species (ROS), impairs insulin-like growth factor-1, and induces stress intracellular kinases that inactivate the translocation of glucose transporter 4 (GLUT4), thus hindering glucose uptake. In addition, fatty acid oxidation-generated ROS dysregulate the Phosphoinositide 3-kinases (PI3K)-AKT signals pathways, which are essential in glucose uptake [17,18]. Moreover, obesity triggers the S-nitrosylation of lysosomal proteins, which leads to lysosomal failure and defective liver autophagy, resulting in an accumulation of defective lipids and misfolded proteins, which eventually increases the ROS production from the ER and mitochondria [19]. Inflammatory cytokines cause insulin resistance, which is characterized by impaired insulin release and the disruption of insulin signal transduction, with the ultimate result of glucose imbalances [20]. In white adipocytes, saturated fatty acids, such as palmitate, induce insulin resistance by promoting the production of ROS, proinflammatory molecules, and apoptosis, which inhibit IRS-1, PI3K, and Akt signaling [21]. The unchecked high blood sugar level is associated with the depletion of the body’s antioxidant defense system and the subsequent generation of reactive oxygen species (ROS). Muscles generate ROS primarily through the mitochondrial respiratory chain and from xanthine oxidase. Moreover, the hyperglycemia-related metabolic processes that contribute to oxidative stress may include glucose oxidation, protein glycation, and lipid peroxidation. In the vasculature, sugars interact covalently with hemoglobin proteins, resulting in glycation end products (AGEs) and nitric oxide (NO). Acute oxidative stress may temporarily increase skeletal muscle glucose uptake [22]. Nevertheless, the uncontrolled production of ROS damages the beta cells in the pancreas, which triggers a vicious cycle of diabetes-associated complications [17,23,24,25]. Due to the intrinsic production of ROS and the limited antioxidant system, pancreatic beta cells are extremely vulnerable to oxidative stress-related damage [26].

It is obvious that physical activity is one of the effective methods to control glucose homeostasis through increasing glucose uptake by a substantial margin. The skeletal muscles are considered the primary driving force of energy expenditure and insulin-mediated glucose uptake during physical activity, which is critical in maintaining glucose homeostasis. Studies show that remedying insulin resistance in the skeletal muscle alone restores glucose homeostasis in the whole body when it is the primary defect [27]. Untreated chronic insulin resistance in the skeletal muscles leads to an irreversible failure in pancreatic β cells. The progression of insulin resistance in the skeletal muscles presages the onset of pancreatic β cells failure or symptomatic type 2 diabetes over several years [27,28]. In the skeletal muscle, insulin-mediated glucose uptake is enhanced through the translocation of GLUT4 following a cascade of signal transduction. Insulin-mediated glucose uptake in the skeletal muscle is profoundly susceptible to insulin resistance, which significantly contributes to obesity-related insulin resistance and type 2 diabetes. The insulin receptor (IR) and its close family member, the insulin-like growth factor-1 receptor (IGF-1R), trigger the activation of PI3K/AKT in the skeletal muscle, which accelerates glycolysis, glycogenesis, and protein synthesis. AKT and IRS adaptor proteins have been shown to diminish insulin-driven glucose uptake in AKT-knocked out or knockdown mice, whilst the overexpression of AKT increases glucose uptake [29]. When insulin is activated, AKT phosphorylates AS160, which releases GLUT4 from storage vesicles into the plasma membrane, enabling glucose transport [30,31]. Furthermore, the activated AKT (Gag AKT) induces glycogen synthesis by stimulating glycogen synthase (GS) in the skeletal muscles, which redirects glucose-6-phosphate’s fate and inhibits glycogen synthase kinase-3 (GSK-3), as demonstrated in the L6 myotubes [27,28]. Moreover, it is believed that insulin stimulates protein synthesis and speeds up mRNA translation in the skeletal muscle by regulating the initial stages in the translation process. Activating rapamycin complex 1 (mTORC1) through AKT modulates a series of downstream effectors that are phosphorylated to increase protein synthesis, including ribosomal S6 protein kinase-1 (S6K1) and eukaryotic translation initiation factor-4E (eIF4E) binding protein-1 (4E-BP1) [29,30,31].

Similarly, the insulin released during sugar ingestion triggers hepatic IRS phosphorylation to regulate glucose and lipid metabolism. Following IRS activation, PI3K generates phosphatidylinositol-3,4,5-triphosphate (PIP3) by phosphorylating PIP2 [32,33]. Due to this activation, Pyruvate Dehydrogenase Kinase 1 (PDK1) phosphorylates Akt at Thr308 through PIP3. Furthermore, mTORC2 activates Akt by phosphorylating Ser473. The activated Akt controls many of the metabolic processes in the liver, such as glycogen synthesis, glucose oxidation, glycolysis, and lipid metabolism. For instance, activated Akt regulates glycogen synthesis in the GSK3-mediated and independent pathways. Moreover, in the phosphorylation of forkhead box protein O1 (FoxO1), Akt inhibits part of the glucokinase genes and gluconeogenic transcription factors such as G6Pc and Pck1. While FoxO1 is shut off by Akt, MTOC1 is activated by Akt, in turn activating the lipogenic genes and promoting lipogenesis [34,35,36]. Although the amount of glucose taken up by adipose tissues is relatively small, the literature shows that adipose GLUT4 levels are associated with insulin sensitivity. The entry of glucose into the adipose tissue triggers de novo lipogenesis and the formation of branched fatty acid esters of hydroxyl fatty acids (FAHFAs) through the carbohydrate response element binding protein (ChREBP). FAHFA increases the insulin-mediated adipocyte’s glucose uptake [37,38,39,40,41,42,43].

## 3. Plant Nutraceuticals

The term nutraceuticals encompass functional foods, fortified foods, fiber, plant extracts, vitamins, minerals, and amino acids. In traditional medical practices before the development of Western medicine, herbal remedies were used, as evidenced by ancient Sumerian clay slabs from Nagpur, the Chinese book on roots and grass by Shen Nung circa 2500 BC, and many more artifacts [44]. Nutraceuticals are becoming increasingly popular, particularly in Western civilization, despite the criticism of the lack of clinical evidence, safety concerns, mythological efficacy, and quality concerns [45,46,47]. The World Health Organization (WHO) developed a traditional medicine strategy in 2014–2023 to harness the potential contributions of traditional medicines to human health and the promotion of its safe and effective use [48]. Phytotherapeutics are increasingly being combined with synthetic drugs to treat certain diseases in modern medicine. However, due to the complexity of the synthesis and purification of active ingredients, only 50% of the active ingredients in plants can be synthesized into pharmaceuticals. Moreover, the accumulation of phytochemicals is perpetual as it is influenced by biotic and abiotic factors such as light, temperature, soil water, soil fertility, and salinity to enhance plant survival [49]. It is apparent that the health benefits of non-purified ingredients can be realized in their natural state [50]. In this review, papaya and seaweed are examined for their antidiabetic effects and the mechanisms by which they normalize blood glucose in various hyperglycemic or/and diabetic models. Further, we expectantly postulate the possibility of combining antidiabetic bioactive compounds to enhance and potentiate their therapeutic effect against prediabetic or diabetic patients. 

## 4. Antihyperglycemic Properties of *Carica papaya*

The papaya plant is a species of *Carica papaya* (*C. papaya*), which is in the family of Caricaceae. It was originally native to South America and is now widely cultivated throughout the world. The *Carica papaya* tree grows to a height of 5–10 m and consists of a single unbranched stem. Each leaflet has seven large palmately lobed leaflets with an approximate diameter of 50 to 70 cm. There is a wide range of papaya products available in the marketplace today, including jams, sweets, and pulp—and other parts of the plant (leaves and seeds) are used in the form of tea and powder to maximize the nutritional value [51]. Various parts of the papaya tree have been shown to exhibit anti-hyperglycemic properties. It was demonstrated that aqueous and ethanol extracts from papaya leaves effectively controlled hyperglycemia in experimental mice treated with diabetic-induced drugs, alloxan, and streptozotocin (STZ) [52,53,54,55]. In alloxan-induced diabetic rats (180 mg/kg), extracts were administered at low or high doses for three or seven days, along with metformin in low and high doses of 50 mg/kg and 100 mg/kg body weight, as well as glimepiride in low and high doses of 0.2 and 0.4 mg/kg, respectively. Papaya leaf extracts at 5 mg/kg body weight produced similar results as glimepiride (0.2 mg/kg) and metformin (50 mg/kg), although with a delayed onset of effect. In contrast to glimepiride monotherapy, papaya leaf extract given together with high doses of glimepiride significantly (*p* < 0.01) increased glimepiride’s onset effect. Furthermore, the reduction in blood glucose at 24 h was highly significant (*p* > 0.001), as, except for the low glimepiride-low papaya leaf combination, the effect extended to 72 h, demonstrating that papaya leave extract can interact directly with the cells or in a complementary manner to induce hypoglycemic effect [56]. Further research is needed to determine the interaction between papaya juice and pharmaceutical drugs, as well as their toxicity and level of efficacy. Elsewhere, two groups of normal pancreatic islets were examined for the effects of papaya leaf extract on insulin-producing islets [57]. Papaya leaf extract was incubated with STZ concurrently in the first group, whereas STZ was added five days after the papaya leaf extracts in the second group. The first treatment significantly (*p* < 0.05) produced more insulin and protected against STZ-induced damage compared to the cells that were simultaneously treated with STZ and papaya leaf extracts. In addition, the cells that were treated with papaya extract alone produced 22% higher amounts of insulin compared to the negative control. Furthermore, papaya leaf extract reduced the glycemic levels (344.5 mg/dL to 122.2 and 113.25 mg/dL with extract doses of 31 and 62 mg/kg, respectively) in STZ-induced diabetes rats and preserved the pancreatic islet integrity [57].

Papaya exhibits protective properties against diabetes-induced beta cell damage, but not regeneration, which is usually a pathological condition in type 1 diabetes. Male Wister rats induced with Type 1 diabetes were hypoglycemic after leaf and seed extracts were administered. These extracts also protected the rats against diabetic-induced liver and kidney damage. The antihyperglycemic effect of the seed extracts was 76% greater than that of the leaf extracts, suggesting that the seed extracts might be more potent [58]. Interestingly, *C. papaya* root extract significantly reduced the fasting plasma glucose level of diabetic rats by 30.95% [59]. Glycated hemoglobin (A1C) decreased to 5.34% in alloxan-induced diabetic male albino rats fed *Carica papaya* green pulp for 28 days, compared with 9.56% in their untreated counterparts and 3.9% in the normal control group; in addition, green pulp also showed significant hypoglycemic effects [59]. The phytochemicals derived from ethyl acetate seed extracts and aqueous extracts of green papaya fruit significantly diminish postprandial hyperglycemia and manifest antioxidant properties with high inhibitory effect (IC_50_) to α-amylase and α-glucosidase enzymes [60,61]. Moreover, fermented papaya improved the membrane potential of platelets from individuals with T2DM through restored Na^+^/K^+^-ATPase activity, increased fluidity of the membrane, and improved SOD activity, thus preventing ROS-related diabetes [62]. In a clinical trial, the administration of 6 g fermented papaya preparation (FPP) per day for 14 weeks facilitated organ recovery by limiting ROS production. A comparison between the sample and the control group revealed a significant reduction in oxidative stress-induced inflammation, a significant improvement in the LDL/HDL ratio, and a significant decrease in uric acid levels [63]. Although the literature has shown that all the parts of *C. papaya*, except mature fruit, may have antihyperglycemic properties, little is known about the specific bioactive compounds found in the crude extracts. In addition, papaya’s therapeutic claims will need to be validated by consistent and reproducible clinical data. 

## 5. Bioactive Compounds in *C. papaya*

The phytochemicals, minerals, vitamins, fatty acids, and fibers in *C. papaya* are implicated in its antihyperglycemic properties. There are also significant differences in the chemical composition and distribution of the bioactive compounds across the leaves, pulp, seed, and fruit [51]. Steroids and quinones dominate the phytochemical metabolites detected in chloroform leaf extracts [64,65]. On the other hand, polyphenolic compounds, including flavonoids, saponins, pro-anthocyanins, tocopherol, and benzyl isothiocyanate, have also been detected in papaya leaf extracts [66]. Additionally, high levels of beta-carotene (up to 888 IU/100 g) have been reported in the fruits, while the seeds have exhibited high levels of fatty acids, glycosylates, tocopherols, cryptoxanthin, and precursors of Vitamin A [67,68]. At present, animal experimental studies have demonstrated papaya’s antihyperglycemic effect, which has been attributed to its bioactive compounds as shown in Table 1 below. However, there are no studies/evidence directly linking the specific implicated bioactive compounds to the reported effects. Therefore, it will be worthwhile to investigate the molecular mechanism behind papaya’s antihyperglycemic effect in its crude or purified forms.

## 6. Therapeutic Effects of Seaweed

Seaweed distribution is somewhat global, including tropical waters and cold polar waters. Similarly, to papaya, it is not only an important food source, but also a potential medicine. The color of seaweed determines its category: brown seaweed (phylum Ochrophyta), green seaweed (phylum Chlorophyta), and red seaweed (phylum Rhodophyta) [72]. Agar, carrageenan, alginates, and other polysaccharides obtained from seaweed are valuable resources for the pharmaceutical and food industries. Agar extracts have been used as thickeners and gelling agents for generations. Asian cuisines have also used edible seaweed species as ingredients, such as in Japanese condiments, seasonings, and sushi wrappers [73,74,75]. Due to environmental stress, seaweed releases secondary metabolites that produce bioactive molecules with unlimited therapeutic potential. Many of these bioactive components have been shown to have therapeutic effects on inflammation, cancer, diabetes, and oxidative stress [74].

## 7. Seaweed Inhibitory Effect on α-Amylase and α-Glucosidase

The bioactive compounds in seaweed, such as polyphenols, carotenoids, vitamins, phycobilins, phycocyanins, Fucoxanthin, Octaphlorethol A, and polysaccharides, have been shown to protect humans from a variety of diseases [3,76]. In addition to vitamins, minerals, and fiber, edible seaweed also contains unsaturated fats, dietary fibers, and trace minerals. With its diverse nutrients, seaweed is becoming increasingly popular for managing diabetes, obesity, and weight gain around the world [77]. A Korean study found that men who consumed *Porphyra yezoensis* and *Undaria pinnatifida*, a species of seaweed plant, as part of their diet were less likely to develop diabetes [78]. Several seaweed bioactive compounds have been investigated for their antidiabetic properties by examining how they affect glucose digestion and absorption from the gut to the peripheral tissues. In studies with Streptozotocin-induced diabetic rats, the seaweed species *Ascophyllum nodosum* almost normalized fasting plasma glucose and regulated glucose spikes during an oral sucrose tolerance test. The intestinal glucose uptake enzymes α-amylase and α-glucosidase were inhibited by the phenolic compounds extracted from *A. nosodsum* at 80 °C, with IC_50_ values of 1.34 and 0.24 µg, respectively, compared to acarbose, which had values of 0.68 and 0.37 µg [79,80]. In addition to inhibiting α-amylase and α-glucosidase, *Sargassum hemiphyllum* extracted from acetone combined with 25 mg/mL of glibenclamide increased insulin secretion in RIN-5F rat beta-cells compared to cells treated with 50 mg/mL of glibenclamide alone [81]. Amylase and glucosidase inhibitory properties have also been demonstrated in several other studies involving other species of brown seaweed extract, such as *Undaria pinnatifida*, *Sargassum serratifolium*, *Sargassum heiphyllum*, *Alaria marginata*, and *Fucus distichus.* Polysaccharides, phlorotannins, plastoquinones, and phenols are among the bioactive substances implicated in this effect [81,82,83,84,85,86,87]. The pure form of fucoxanthins, one of the most characterized compounds from *Undaria pinnatifida* extracts, was found to exhibit inhibitory effects on α-glucosidase activity [88]. Purified forms of Fucoidans from *Turbinara conoides*, *Turbiniaria ornate*, *Sargassum wightii* have also been found to possess inhibitory effects on both α-glucosidase and α-amylase [89]. Furthermore, research on red seaweed has also found that ethanolic extracts and algae-derived peptides (Gly-Gly-Ser-Lys and Glu-Leu-Ser) inhibit α-amylase enzyme [90,91]. The antidiabetic potential of green algae has been relatively underexplored, although *Halimeda macroloba* was shown to inhibit α-amylase enzyme activity, and using RubSCo protein in silico analysis of the peptide from *Ulva lactuca* showed its inhibitory activity against α-glucosidase [92,93]. 

## 8. Seaweed and Glucose Metabolism: Molecular Interactions

Increased insulin sensitivity may be a more effective approach to controlling type 2 diabetes. For instance, in a diet-streptozotocin rat model, *Sargassum polycystum* extracts decreased the fasting plasma glucose, reduced dyslipidemia, and ameliorated the oxidative stress without altering the insulin levels, suggesting an improvement in the insulin sensitivity [94]. Similarly, fucoidan reduced the fasting plasma glucose levels, increased the insulin sensitivity, enhanced the liver antioxidant defense system and blunted the inflammatory markers in a non-alcoholic fatty liver disease model [95]. Fucoidan and fucoxanthin independently reduced the fasting plasma glucose and triggered glycogen synthesis in the liver of the T2DM mouse model. However, only fucoxanthin upregulated the expression of insulin-sensitizing molecular pathway players (RS-1, GLUT-4, and PPAR-γ) in the adipose tissue, whilst the combination of fucoidan and fucoxanthin had a greater effect in the upregulation of insulin-sensitizing pathway molecules [96,97]; these results demonstrate that by combining bioactive compounds, either reinforcement or synergy can enhance their effectiveness. A partial improvement in insulin sensitivity by fucoxanthin has also been attributed to the downregulation of inflammatory adipokines, such as monocyte chemoattractant protein-1 (MCP-1), tumor necrosis factor (TNF-), interleukin-6 (IL-6) and plasminogen activator inhibitor-1 mRNA in the white adipose tissue (WAT) of KK-Ay mice. In addition, fucoxanthin also reduced the expression of inducible nitric oxide synthase (iNOS) and cyclooxygenase-2 (COX-2) mRNA in palmitic-induced macrophages [97]. It is suggested that Fucoxanthin may improve insulin uptake by inhibiting inflammation in the WAT. Moreover, a study of C2C12 myoblast cells showed that Ecklonia cava’s antihyperglycemic effect in Type 1 diabetic rats was mediated partly by activating the AMPK and Akt pathways [98]. Octaphlorethol extracts were shown to induce glucose uptake in L6 myoblasts through AMPK and the PI3/Akt signaling pathway [99]. In addition to the studies shown in Table 2, more studies with a variety of in vitro and in vivo models are needed to understand the effects of a variety of bioactive compounds at the molecular level.

There is an apparent need for extensive studies on red and green seaweed that may add to the pool of bioactive compounds already identified in brown seaweed. This is because the data on red and green seaweed has not been as extensively examined as the data on brown seaweed.

## 9. Enhancing Therapeutic Effects of Papaya and Seaweed

**Figure 1:**
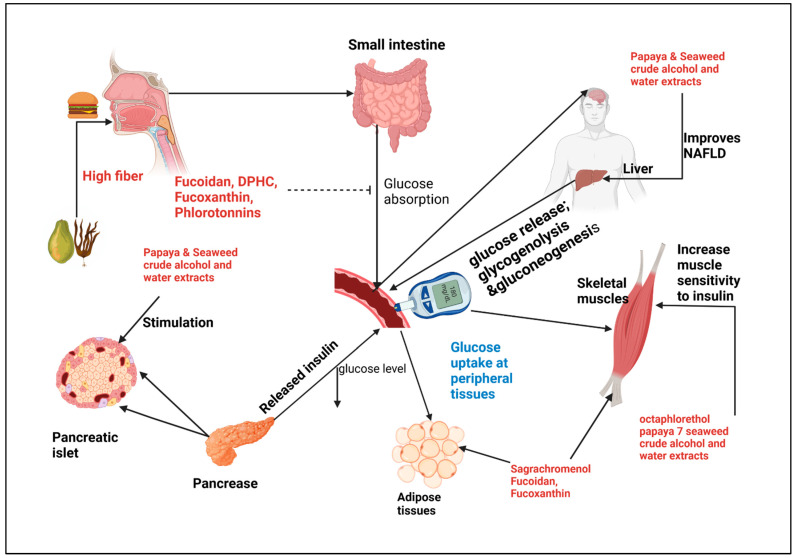
Schematic representation of papaya and seaweed antihyperglycemic mechanisms. At digestion consumption of high fiber Papaya and seaweed slows blood sugar increase, intestinal absorption inhibitor of α-amylase and α-glucosidase are inhibited by fucoidan, DPHC, Fucoxanthin, phlorotannin. In the peripheral organs insulin mediated uptake is activated by Octaphlorethol, Fucoidan, sagrachromenol, alcohol and water crude extracts. Crude extracts stimulate pancreatic islet and improve hepatic NAFLD.

**Figure 2:**
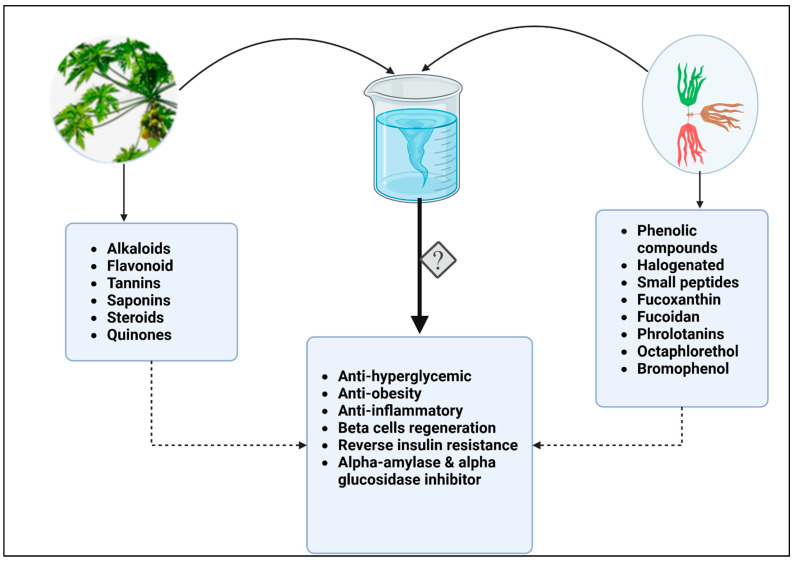
Combined bioactive compounds from papaya and seaweed may have higher potency against diabetes. While papaya leaves, fruit and roots contain alkaloids, flavonoids, tannins, saponins, steroids, and quinones, some of the active compounds in brown, red, and green seaweeds include phenolic and halogenated compounds. Phlorotannins, fucoxanthins, and octaphlorethol, small peptides, are also antidiabetic, anti-obesity, and anti-inflammatory, as well as acting as beta cells regenerators and inhibiting glucose metabolic enzymes. A combination of bioactive compounds during extraction, or in their crude form may have synergistic effect and increase therapeutic potential of these natural products.

Traditional herbal medicine was enhanced with a combination of different herbs, while Western medicine had been influenced by monotherapeutic approaches. The paradigm shifts to the polytherapeutic approach, where multiple drugs are used to maximize drug efficacy, has stirred up scholarly interest in herbal medicine combinations [110]. Plant survival and the production of useful natural products are enhanced by secondary metabolites in response to abiotic environmental factors. Figure 1 illustrates how papaya and seaweed extracts, as well as purified bioactive compounds, can affect glucose metabolism. Studies have shown that herbal combinations can have a synergistic effect and have been repurposed to tackle emerging complications such as COVID-19 in recent years [110,111,112,113]. As natural products have a broader and more long-term physiological effect than pharmaceutical products [107], the combination of papaya and seaweed bioactive compounds in their purified form or crude extracts (Figure 2) may be more effective in the management of diabetes and the related complications, which include hyperglycemia, dyslipidemia, inflammation, and obesity, in comparison to the short-term pharmaceutical products. Additionally, these combinations could have synergistic or complementary effects and they can be consumed with minimal side effects, as opposed to synthetic drugs. Several seaweed antidiabetic compounds have been isolated and their mechanisms of action have been elucidated, as shown in (Table 2). In contrast, papaya’s antidiabetic effects are attributed to crude extract mixtures rather than a specific bioactive component. Furthermore, the papaya tree’s fruits, seeds, leaves, and pulp exhibit varying anti-diabetic properties (Table 1) [70,71]. The formulation of the cocktail could be achieved in several ways based on the efficacy tests; however, it should be noted that these products should be kept in their natural form as intense purification reduces the pharmacological effects of bioactive compounds [114].

## 10. Summary

The cause of T2DM is complicated by genetic and non-genetic factors, such as a sedentary lifestyle and diet. This leads to an imbalance in the energy intake and expenditure of the body. Among the bioactive compounds extracted from papaya and seaweed are vitamin complexes, polysaccharides, phenolic compounds, essential fatty acids, flavonoids, fucoidan, and phlorotannin, among others. The modulation of α-amylase and α-glucosidase activity through these bioactive compounds can control # intestinal glucose uptake, which enhances the tissue insulin sensitivity and glucose uptake of the skeletal muscle and adipose tissue via targeting the AMPK/Akt/GLUT4 signaling pathway. Significant improvements in controlling glucose homeostasis and increasing insulin sensitivity can be achieved by managing weight gain and obesity, especially abdominal obesity, which has been closely linked to diabetes. Although physical activity, highly depending on the skeletal muscle mass and physiology of patients, is important for controlling glucose homeostasis, skeletal muscle loss is frequently associated with diabetes. Long-term insulin malfunctions in both T1DM and T2DM increase patients’ catabolism, resulting in serious muscle loss, which has a direct impact on their capability of physical activity. Furthermore, papaya and seaweed vitamin complexes help in mitigating the condition by increasing the expression of antioxidative enzyme networks, reducing ROS production, reducing NO production, and abating inflammation; hence, they exert antidiabetic effects. There is emerging scientific evidence supporting the practice of different herbs that are traditionally combined to enhance their therapeutic effects. The mechanism of each specific active compound from seaweed and papaya is not defined and the research results have mostly been obtained through association analysis. As single-drug targets are shifting to synergistic network pharmacology to optimize drug response [110], using this approach, it may be possible to combine bioactive compounds from various sources of natural products to explore the therapeutic potential of nutraceuticals. Combining papaya and seaweed using empirically studied bioactive compounds will enhance an effective cocktail against diabetes and the related complications, which include hyperglycemia, hyperlipemia, inflammation, and obesity. Further research into the molecular mechanisms and their efficacy in animal models and clinical trials will be necessary for the broad applications of diabetes prevention and management.

## Figures and Tables

**Table 1:** Antidiabetic properties of crude papaya extracts in experimental animal models.

Diabetic Model	Papaya Part	Papaya Dosage	Effect	Mechanism	References
Alloxan-induced diabetic rats; Dosage: 90 mg/kg	Root	Diabetic rats: 500 mg/Kg root aqueous extract Duration: 21 days	Reduction of 30.95% in sugar levels after 7 days	The role of antioxidants may be involved	[69]
Alloxan induced Rats (120 mg/kg)	leaves	*C. papaya* aqueous extract 100, 200 and 400 mg/kg	400 mg/kg was effective in controlling glucose levels after 21 days	Unknown	[70]
Alloxan-induced rats (150 mg/kg)	leaves	*C. papaya* ethanolic extract 250 and 500 mg/kg	Dose-dependent antihyperglycemic effect 43.8% and 51.1%, respectively, after 21 days	unknown	[52]
Streptozotocin induced rats (60 mg/kg)	leaves	Chloroform-extracted was administered at 31, 62, and 125 mg/kg for 21 days	62 mg/kg was effective in reducing high sugar levels by 67%	Steroid-mediated effect	[64]
Streptozotocin induced rats (60 mg/kg)	leaves	Aqueous extracts were dispensed at doses of 0.75, 1.5, and 3 g/100 mL for 30 days	0.75 and 1.5 g/mL treatment significantly reduced sugar levels in diabetic rats	Hyperstimulation of β-cells	[55]
Streptozotocin induced mice (60 mg/kg)	leaves	Ethanol extract of *C. papaya* and *P. amarylifolius* in a dose of 100 mg/kg each for 6 days	Both treatments significantly lowered the sugar level in the diabetic model	Phytochemicals-mediated	[71]

**Table 2:** Anti-diabetic bioactive compounds in seaweed as demonstrated in animal models with proposed mechanisms of actions.

Seaweed Type	Species Name	Bioactive Compound	Experimental Model	Proposed Mechanism	References
Brown Seaweed	*Ascophyllum nodosum* and *Fucus vesiculosis*	polyphenols, Polysaccharides, fatty acids, fucoidan	non-alcoholic steatohepatitis (NASH) mouse model	α-amylase and α-glucosidase inhibitor	[100,101]
Brown Seaweed	*Ishige okamurae*	Diphlorethohydroxycarmolol (DPHC)	Type-1 diabetic mice	α-amylase and α-glucosidase inhibitor	[102]
Brown Seaweed	*Hizikia fusiformis*	Fucoxanthin, fucosterol	In vitro model RAW 264.7 cells	α-glucosidase inhibitor	[103]
Brown Seaweed	*Turbinara conoides*	Fucoidan	Invitro and in silico	α-amylase and α-glucosidase inhibitor	[87]
Brown Seaweed	*Ecklonia maxima* (Osbeck) Papenfuss	phlorotannins	In vitro assay	Antioxidant and α-glucosidase	[104]
Red Seaweed	*Symphyocladia latiuscula*	bromophenols	In vitro assay Rat lens	Inhibitory effect on PTP1B	[105]
Green Seaweed	*Capsosiphon fulvescens*	CH_2_Cl_2_, EtOAc, and *n*-BuOH extracts	In vitro assay	Inhibitory effect of Aldose reductase and AGEs	[106]
Brown seaweed	*Ecklonia cava*	Methanolic extract	Type-1 diabetic rat and C2C12 myoblasts	Activate MPK/ACC and PI-3 kinase/Akt signal	[97]
Brown seaweed	*Ishige foliacea*	Octaphlorethol A (OPA)	L6 rat myoblast cells	PI3-K/Akt and AMPK activation	[98]
Brown seaweed	*Ecklonia cava*	Crude Extract (phlorotannin)	Clinical trials	Unspecified (reduce postprandial hypeglycemia)	[107]
Brown seaweed	*Sargassum polycystum*	Ethanolic and aqueous extracts	Type 2 diabetic rat	Increase insulin sensitivity	[93]
Red seaweed	*Laurencia dendroidea*	Acetate and ethanolic extracts	Type 1 diabetic rat and in vitro assay	α-Glucosidase inhibitor, antihypeglycemic and antioxidant	[108]
Green seaweed	*Enteromorpha prolifera*	flavonoid-rich fraction	Type 2 diabetic mice (streptozotocin high fat and sugar diet)	IRS1/PI3K/AKT and inhibition of the JNK1/2 insulin pathway in liver	[109]

## Data Availability

Not applicable.
